# Conservative mechanism through various rapeseed (*Brassica napus* L.) varieties respond to heavy metal (Cadmium, Lead, Arsenic) stress

**DOI:** 10.3389/fpls.2024.1521075

**Published:** 2025-01-14

**Authors:** Lingyu Li, Zhanhuang Fan, Qingqin Gan, Gang Xiao, Mingbao Luan, Rilong Zhu, Zhenqian Zhang

**Affiliations:** ^1^ College of Agriculture, Agricultural University of Hunan, Changsha, China; ^2^ China Energy Conservation Land (Hangzhou) Environmental Restoration Co., LTD., Hangzhou, China; ^3^ Institute of Bast Fiber Crops, Chinese Academy of Agricultural Sciences, Changsha, China; ^4^ College of Chemistry and Chemical Engineering, Hunan University, Changsha, China

**Keywords:** rapeseed, heavy metal, peroxidase, ABC transporters, WH23

## Abstract

**Introduction:**

Heavy metal soil pollution is a global issue that can be efficiently tackled through the process of phytoremediation. The use of rapeseed in the phytoremediation of heavy metal-contaminated agricultural land shows great potential. Nevertheless, its ability to tolerate heavy metal stress at the molecular level remains unclear.

**Methods:**

Here, with 7-day seedlings as raw materials, we investigated physiological and biochemical indexes, analyzed the transcriptome sequencing for different treated materials (control, 50×, and 100×), combined with the results of transcriptome and proteome sequencing of the near-isogenic lines (F338 and F335) to reveal the response mechanism to heavy metal stress. Due to oxidative stress response caused by heavy metal stress, there are heavy effects on the emergence of rapeseeds and the growth of seedlings. Although rapeseed can alleviate oxidative stress by enhancing the enzyme activity, especially peroxidase in the oxidation system, this process has its limits. Rapeseed plants activate antioxidase, transport enzymes, and biological regulation to cope with heavy metal stress. Among these responses, peroxidase, ABC transporters, and abscisic acid are particularly significant in this process.

**Results and discussion:**

Based on this study, we identified a breeding material with high adsorption capacity for heavy metals, which contributed to the research on resistance breeding in rapeseed. The results of this study may be useful to alleviate heavy metal soil pollution and tackle edible oil shortages in China.

## 1 Introduction

Heavy metal pollution mainly refers to excessive content of heavy metals (such as Cd and Pb in water, soil, and air. According to the National Soil Pollution Survey Bulletin issued by the Ministry of Environmental Protection and the Ministry of Land and Resources in 2014, the spot exceedance rates of eight heavy metal elements in soil, namely cadmium (Cd), mercury (Hg), arsenic (As), copper (Cu), lead (Pb), chromium (Cr), zinc (Zn), and nickel (Ni), were 7.0%, 1.6%, 2.7%, and 2.1%, respectively, 1.5%, 1.1%, 0.9%, 4.8% (Li et al., 2024). In China, due to large population and limited amount of arable land, it is necessary to use agricultural land with mild heavy metal pollution (Kopittke et al., 2019). However, heavy metals affect the environment, plant growth and development, and human health (Dhaliwal et al., 2021; Gong et al., 2022). Determining the means to repair and use heavy metal contaminated soil has become important, especially in developing nations (Jawed et al., 2020). At present, the main restoration methods of heavy metal contaminated soil include physical and chemical remediation and bioremediation. Physical remediation includes soil washing, surface capping, soil replacement, etc (Feng et al., 2020; Ahmed et al., 2021), and chemical remediation includes vitrification technique, chemical leaching, ion-exchange, etc (Wang et al., 2021; Yang et al., 2021). Both of these two remediation methods have same problems, such as high remediation cost and difficulty in applying large-scale soil remediation (Rajendran et al., 2022; Dhaliwal et al., 2020; Jaiswal and Shukla, 2020). Bioremediation refers to theuse of plants and microorganisms to reduce the heavy metal content in soil (Khoo et al., 2021). Compared with physical and chemical remediation, bioremediation, especially phytoremediation, is more suitable for large-area soil remediation (Kong and Glick, 2017; Rajendran et al., 2022) for its low cost. Phytoremediation refers to the use of plants and related microorganisms to reduce the potential impact of heavy metals in the environment, and mainly includes phytovolatilization, and phytoextraction (Sarwar et al., 2017; Muthusaravanan et al., 2018). Compared with other remediation methods, phytoremediation is more suitable for large-scale soil remediation because of its low planting cost, large planting area and less pollution. Plant species, soil features, and heavy metal status are the main factors affecting the phytoextraction rate (Li et al., 2012; Budzyńska et al., 2021). There are mainly two different plant types used in phytoextraction: hyperaccumulator plants with relatively low biomass production, and plants with higher aboveground biomass production but lesser metal accumulation (Li et al., 2022). The phytoextraction involves five major steps: Metal mobilization in rhizosphere, metal ion uptake by plant roots, translocation towards aerial plant parts, metal sequestration in plant tissues, and heavy metal tolerance (Ali et al., 2013). Following accumulation, heavy metals will be transferred to food along the food chain and may endanger human health (Bolan et al., 2014). Therefore, it is important to select suitable plant species to rehabilitate heavy metal contaminated soil which can be used post-harvest without endangering human health.

In China, rapeseed is one of the largest oil crops and an important source of edible oil. USDA data show that the consumption of rapeseed oil in China in 2020/2021 accounts for 20% of all vegetable oil consumption (Zhang R, et al., 2020). At present, the production, import and export of rapeseed and rapeseed oil is an important part of China’s oilseeds and fats, which is related to the supply and demand balance of China’s oilseed and fats market. Many researchers have shown that rapeseed can be used as an excellent plant for remediation of heavy-metal contaminated soil (Yu et al., 2014; Zeng et al., 2020; Yuan et al., 2020; Cao et al., 2021). Studies have shown that oilseed rape can easily absorb heavy metals when grown in heavy metal-contaminated soil, and is characterized by high biomass and rapid growth, thus attracting the attention of scholars (Liang and Huang, 2021). Surprisingly, rapeseed oil did not contain heavy metals even when rapeseed plants were used to repair heavy-metal contaminated soil (Yuan et al., 2020). Therefore, rapeseed can not only repair the soil, but also alleviate the limitation in the edible oil supply of edible oil supply in China. Many studies have focused on improving the efficiency of rapeseed for adsorbing heavy metals through external measures such as nitrogen fertilizers, rhizosphere interactions, and organic acids, etc (Zeng et al., 2020; Qiao et al., 2020; Li et al., 2021). However, the mechanism by which rapeseed responds to heavy metal toxicity has not yet been fully elucidated, which, to a certain extent, hinders the application of rapeseed in the remediation of heavy-metal contaminated soil.

The main aim of this study was to reveal the mechanism of the response of rapeseed to heavy metal stress by transcriptome and proteomics. In addition, in this study we tried to screen out suitable rapeseed varieties or materials for remediation of heavy metal contaminated soils.

## 2 Materials and methods

### 2.1 Experimental design

Initially, a total of 127 different rapeseed genotypes were used to screen materials (**Supplementary Table S1**). The heavy metals included Cd, Pb, and As, and three concentrations were used, i.e., 10 times, 50 times, and 100 times the concentration of the corresponding heavy metal safety standards in the water (**Supplementary Table S2**). This research was carried out according to the following scheme: (a).50 seeds of each material were soaked in a 10× concentration of the heavy metal mixed solution for 12 h, then placed these seeds in a Petri dish containing filter paper (the filter paper was wetted with the same concentration heavy metal solution) and used the same concentration heavy metal solution every day. Water was replenished on one occasion, and the filter paper was kept moist. Culturing was continued for 7 d under the following conditions: Temperature 25°C, light 16 h/dark 8 h, and average light intensity 2455l lux. The germination rate was determined, the material with a germination rate > 80% was used for the 50× treatment; (b) physiological and biochemical indexes and heavy metal contents of 7-day seedlings from 18 materials were determined; (c) transcriptome sequencing of seedlings treated with different concentrations for 7 d on the same material was conducted; (d) transcriptome and proteome sequencing were performed on the seedlings treated with 100× concentration for 7 d on the two materials with significant difference in germination rate and growth condition; (e): different materials were planted in two experimental fields (heavy metal contaminated soil and normal soil), and gene expression and heavy metal contents were determined.

### 2.2 The measurement of antioxidant enzyme system activity

To study the effect of heavy metals on the growth and development of rapeseed, the germination potential, germination rate, emergence rate, activities of superoxide dismutase (SOD), peroxidase (POD), catalase (CAT), and the malondialdehyde (MDA) content were measured. We recorded the germination and growth status of rapeseed seeds at regular intervals every day, and the germination potential, germination rate, and emergence rate were calculated according to the following formula (Zhao et al., 2017).


$${\text{X}}_{\text{gp}}={\text{X}}_{1}/\text{X}\times 100\%,$$

$$\text{Xgr}={\text{X}}_{2}/\text{X}\times 100\%,\text{ and}$$

$$\text{Xer}={\text{X}}_{3}/\text{X}\times 100\%,$$

where

X_gp_: germination potential (%)

X_1_: number of germinated seeds in 3 d

X: number of seeds used in the study

X_gr_: germination rate (%)

X_2_: number of germinated seeds in 7 d

X_er_: emergence rate

X_3_: number of seedlings in 7 d

Before measuring SOD, POD, and CAT activity and the MDA content, it was necessary to configure a buffer and enzyme solution; 0.05 M phosphate buffer was configured by NaH_2_PO_4_·2H_2_O and Na_2_HPO_4_·12H_2_O. The enzyme solution preparation method was as follows: (1). 0.1 g of sample weighed into a mortar, added 0.3 mL of 0.05 M (pH 7.8) phosphate buffer, and then grounded on an ice bath. (2) The homogenate was poured into a 2 mL centrifuge tube and centrifuged at 4 °C for 20 min (10000 r/min). (3) The supernatant was poured into a test tube (centrifuge tube) and stored at 0−4 °C. SOD, POD, and CAT activities were determined according to Yang et al. (2014) and Shi et al. (2010). The MDA content was determined according to Draper et al. (1993).

### 2.3 Measurement of the concentration of C’ Assessment of the concentrations of cadmium, lead, and arsenic in rapeseed plants.

Samples were oven-dried and were then digested with HNO_3_/HClO_4_ mixture (5/1, V/V) at 120°C for 6 h. Cd, Pb, and As concentrations in the digestion solution and soluble fraction were determined using an inductively coupled plasma−optical emission spectrometer (ICPE-9820, Shimadzu, Japan). Plant standard reference materials (GBW (E) 100360) were used for quality control and quality assurance by routine analysis, and blanks were included (Liu, 2012).

### 2.4 Transcriptome sequencing and analysis

WH23 was used as the plant material to analyze the changes in transcription levels in rapeseed under different heavy metal stress (control [CK], 50 times concentration [50×], and 100 times concentration [100×]). Total RNA used for the RNA-Seq was isolated from three seedlings of each different groups. Nanodrop ND-1000 system (Thermo Scientific, USA) and Agilent 2100 (Agilent, USA) was used to detect the RNA integrity and concentration. 15 cDNA library constructed was based on the method of Zhu et al. (2001). Sequencing was performed with an Illumina HiSeq 2500 instrument by Hangzhou Jingjie Biological Technology Co., Ltd. (Hangzhou, China). After removal of reads containing poly-N and low-quality reads, the remaining clean reads were mapped to the reference rapeseed genome using HISAT2 or StringTie from which unigenes were obtained (Kim et al., 2015; Pertea et al., 2015). Unigene expression levels were calculated as fragments per kilobase of exon model per million mapped fragments (FPKM) using the Cufinks program and read counts for each gene were obtained using htseq-count. Gene expression levels in various samples were compared by the DESeq method, with p value < 0.05, fold-change > 2, or fold-change < 0.5, used as thresholds indicating significant differences in gene expression (Anders and Huber, 2010). GO (http://www.geneontology.org/) and KEGG (https://www.kegg.jp/) pathway enrichment analyses of different expression genes (DEGs) were both performed using R, based on a hypergeometric distribution.

### 2.5 Proteome sequencing and analysis

F335 [100×] and F338 [100×] were used as materials to analyze the changes of transcription level and protein level of rapeseed under the stress of heavy metals with the same concentration and different materials. According to Niu et al. (2020), protein was extracted from rapeseed seedlings, and the protein concentration was detected using a BCA protein concentration detection kit (Beyotime Biotechnology, China). The protein was trypsin digested based on Niu et al. (2020). The peptides digested by trypsin were desalted with Strata X C18 (Phenomenex) and then freeze-dried in vacuo. The peptides were dissolved with 0.5 M TEAB, and were labelled according to the instructions of the TMT kit (ThermoFisher Scientific, USA). Peptides were separated by ultra-high performance liquid system, injected into the NSI ion source for ionization, and then entered the Q Exactive™ Plus mass spectrometer for analysis. The working procedures of liquid chromatography and mass spectrometry were conducted according to Jingjie Biological Technology Co., Ltd. (Hangzhou, China). The raw data were processed by the MASCOT engine (Matrix Science, London, UK; version 2.2), and Proteome Discoverer 1.4 software was used to process MS/MS spectra. GO, KEGG, COG, and Praf databases were used to annotate protein functions. Proteins with a P-value ≤ 0.05 and fold-change ≥ 1.3 or ≤ 0.77 were recognized as differentially abundant proteins (DAPs).

### 2.6 Weighted correlation network analysis

In order to find the possible regulatory network of rapeseed in response to heavy metal stress, we conducted Weighted Gene Co-expression Network Analysis (WGCNA) on highly variable genes. First, we only selected genes with FPKM greater than one in all samples for analysis, and used MDA method (Minimum Description Length) to filter out the genes with small changes in different samples, and only retain the first 9000 genes. Then, according to the scale-free network model, the genes were divided into different modules, and the module minimum size was 30 and height cut was 0.25. To identify modules significantly associated with heavy metal stress, the pearson correlation and p-value between each module and different degrees of heavy metal stress were calculated. 15 samples were divided into three groups, namely CK (WH23_CK), low (WH23_50x), and high (WH23_100x, F335_100x and F338_100x). All WGCNA analyses were performed using the WGCNA R package (Langfelder and Horvath, 2008).

### 2.7 Field experiment

To further determine the impact of heavy metal stress on rapeseed in agricultural land, five varieties of rapeseed were planted in two different fields, five varieties of rapeseed were planted in two different fields at a planting cite in Hunan Agricultural University (113。090 E, 28。190 N), respectively. Five rapeseed varieties widely promoted in southern China were used as field experiment materials. Rapeseed were planted in a field, and were transplanted to farmland for FQ (heavy metal exceeding the standard) and FY (heavy metal not exceeding the standard) after the 5−6 leaves period. 60 d after transplanting, fresh weight, dry weight, and heavy metal contents of root and leaves were determined. The determination method of SOD, CAT and POD activities and MDA content was the same as above. Except for the different concentrations of heavy metals in the soil, the two fields had the same growth environment and field management.

### 2.8 Statistical analysis

Data (enzyme activity, heavy metal content, FPKM value, gene expression levels, etc.) reported in the figures are the average values of at least three different measurements. SAS 9.0 was used to determine significance, and different letters (a, b, c…) represent significance at p ≤ 0.05.

## 3 Results

### 3.1 Differences in tolerance of different rapeseed varieties to heavy metals

The ability of rapeseed to germinate and grow into seedlings under heavy metal stress was the first and most important step. Hence, we initially performed a statistical analysis on the germination rate, seedling emergence rate, and various parameters of different rapeseed varieties subjected to heavy metal treatments of varying concentrations. In 10x, the germination potential, germination rate, and emergence rate were 68.37%, 87.80%, and 27.78%, respectively (**Supplementary Table S1**). The germination rates of 103 different rapeseed plants were > 80%; the highest was 100% and the lowest was 0% (**Supplementary Table S1**). Therefore, 103 different rapeseed materials were used in 50×. Subsequently, 88 different rapeseed materials were used in the 100× based on the results of the 50× (**Supplementary Table S1**). In 100×, the germination potential, germination rate, and emergence rate were 67.38%, 77.49%, and 3.48% (**Supplementary Table S1**) respectively; and in the 10×, they were 85.93%, 97.55%, and 34.44%, respectively (88 different rapeseed plants); the biomass weight also changed from 1.78 g to 1.28 g in 100x and 10x. Therefore, the higher the heavy metal concentration, the more affected was rapeseed growth and development. In addition, different rapeseed varieties exhibited different tolerances to heavy metals, whereby some varieties would not germinate in 10×, and others germinated well in 100× concentration.

### 3.2 Differences in antioxidant system and heavy metal content

**Figure 1:**
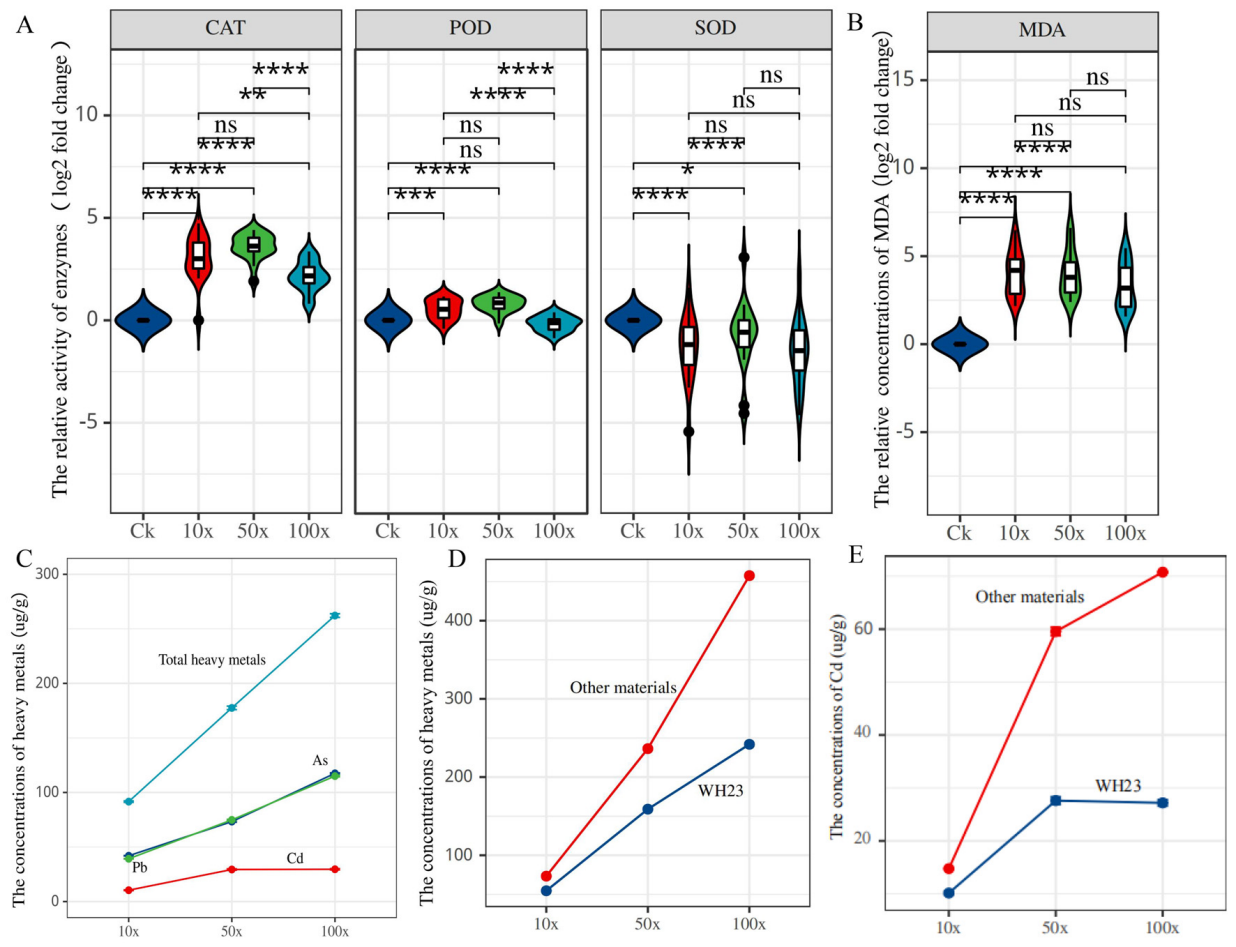
Antioxidant system enzyme activities and heavy metal content in different treatments. **(A)**. the relative activity of CAT, POD and SOD; **(B)**. the relative concentrations of MDA; **(C)** the average concentrations of heavy metal in 18 different varieties; **(D)**. the average concentrations of heavy metal in WH23 and other 17 different varieties; **(E)**. the average concentrations of Cd in WH23 and other 17 different varieties. One asterisk (*): represents a significance level of 0.1. Two asterisks (**): represents a significance level of 0.05. Three asterisks (***): represents a significance level of 0.01. Four asterisks (****): represents a significance level of 0.001.

Based on the results of 100×, 18 different rapeseed varieties were used to determine the SOD, POD, and CAT enzyme activity, and the heavy metal and MDA contents. With increased heavy metal stress, the indicators of CAT,SOD, POD, and MDA all exhibited obvious changes (**Figures 1A**, **B**; **Supplementary Figure S1**). The MDA content reflected the degree of cell membrane damage when exposed to reactive oxygen species (ROS). In the three treatment groups, the MDA content was significantly increased compared with that in the control group, indicating that heavy metal stress damages the rapeseed cell membrane.

We also determined the heavy metals content in rapeseed seedlings of 18 different varieties in the three treatment groups. In the 10× treatment group, the As, Pb, and Cd contents of rapeseed seedlings were 42.96, 3.61, and 10.6 µg/g, respectively, and the total heavy metal content was 56.63 µg/g (**Figure 1C** and **Supplementary Table S3**). In the 50× and 100× treatment groups, the As contents were 76.82 and 125.21 µg/g, respectively, i.e., nearly double and triple, respectively, than that in the 10× treatment group (**Figure 1C**). There has no significant change in the Cd content in the 50× (28.35 µg/g) and 100× (28.19 µg/g) concentration groups. Therefore, Cd adsorption in rapeseed has limited value in terms of phytoremediation. Surprisingly, the amounts of Pb adsorbed in the 50× (56.78 µg/g) and 100× (102.07 µg/g) concentration groups 15 and 30 times that of the 10× concentration group (3.61 µg/g); this was the largest change among the three heavy metals. At 50× and 100×, the total heavy metals adsorption capacities were 161.95 and 255.46 µg/g, respectively, mainly composed of As and Pb (**Figure 1C**). We explored the correlation between measured values, and there was a strong correlation between total heavy metal adsorption and POD activity in the three different treatment groups; the correlation coefficients were 1.00, 0.97 and 0.82 in the 10×, 50×, and 100× treatment groups, respectively (**Supplementary Figure S1E**). There were also other correlations among other indicators in one treatment group, such as Cd and As in the 10× group, and Cd and Pb in the 50× group; however, there has no correlations between all three treatment groups (**Supplementary Figure S1E**). Therefore, within a certain heavy metal concentration, POD activity could reflect the amount of heavy metal absorbed by rapeseed, which could then reflect the heavy metal content in the soil.

Different rapeseed varieties exhibited different abilities to adsorb heavy metals. In the 100× treatment group, the maximum adsorption value of heavy metals was 457.60 µg/g and the minimum was 153.38 µg/g; the difference between different varieties was equally obvious in the 10× and 50× treatment groups (**Supplementary Table S3**). WH23 was an excellent material for adsorbing heavy metals; its total heavy metal adsorption capacities were 73.53, 236.28, and 457.60 µg/g, respectively, in the three treatment groups (**Figure 1D**). In addition, its ability to absorb Cd was much greater than other varieties; the Cd adsorption capacities were 14.80, 59.5, and 70.93 µg/g, respectively (**Figure 1E**). The Cd adsorption capacities of other varieties were 26.52 and 25.67 µg/g in the 50× and 100× treatment groups, respectively. Notably, the amount of Cd adsorbed did not increase with the increase of the Cd concentration in the environment; this indicates that 26.52 µg/g may be the highest adsorption capacity of the other varieties. However, the Cd adsorption value of WH23 in the 100× group was still significantly higher than that in the 50× group, and was obviously different from other varieties. Furthermore, the ability of WH23 to adsorb As and Pb was excellent; the As and Pb adsorption values of WH23 in the 100× group were 216.53 and 170.13 µg/g, respectively. Therefore, WH23 can be used as an excellent rapeseed variety for adsorbing heavy metals, especially for Cd.

### 3.3 Field experiment

**Figure 2:**
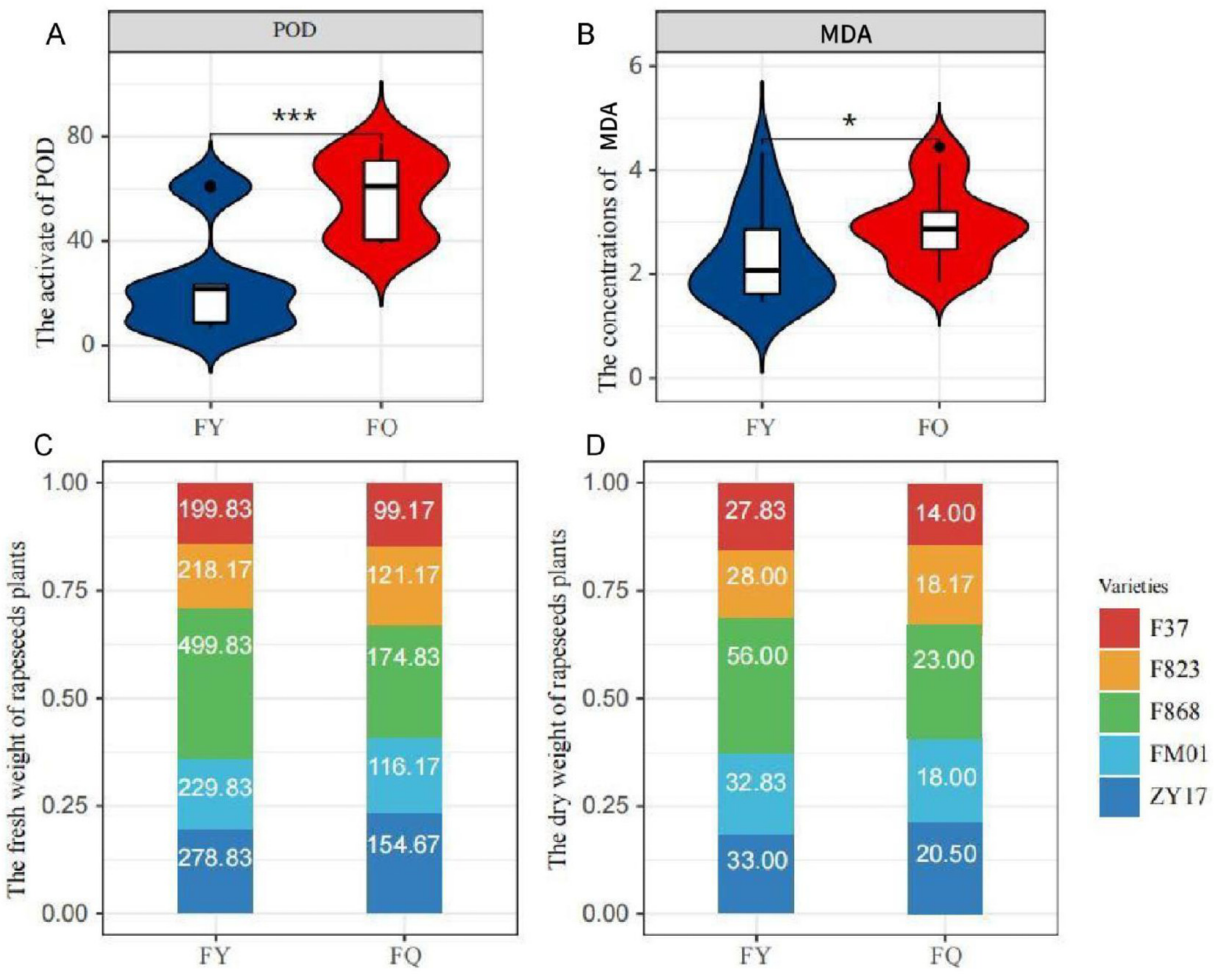
Phenotypes in field experiment.**(A)**. the activity of POD in two different experimental field; **(B)**. the concentrations of MDA in two different experimental field. **(C)**. the fresh weight of rapeseeds plant of two different experimental field; **(D)**. the dry weight of rapeseeds plant of two different experimental field. One asterisk (*): represents a significance level of 0.1. Three asterisks (***): represents a significance level of 0.01.

Plants grown in FQ had significantly higher POD activity and MAD concentration compared to those grown in FY (**Figures 2A, B**), and the CAT activity also had significantly change (**Supplementary Figure S2**). Growth of rapeseed in FY was better than that in FQ, and the fresh and dry weight per plant from FY was almost twice that of plants from FQ. In FY, the highest fresh and dry weight was 499.83 and 56 g (F868), respectively, with average weights of 285.30 and 35.53 g, respectively (**Figures 2C, D**). F868 exhibited the optimal growth in FQ; the fresh and dry weights were 174.83 and 23.00 g, respectively (**Figures 2B, C**). Interestingly, the heavy metal content of FY plants was lower than that of FQ plants, both in roots and leaves. The heavy metal content of FY plants was 9.29 µg/g lower than that of FQ plants, and the contents in root and leaves were 5.80 and 3.49 µg/g (**Table 1**), respectively. Among five varieties, the heavy metal content of F868 was not the lowest; however, F868 had the highest biomass in both fields, and FM01 and ZY17 had lower heavy metal content and biomass. This showed that both varieties and heavy metal content influenced rapeseed biomass. The heavy metal content in leaves was higher (**Table 1**). Therefore, after the roots absorb heavy metals, rapeseed plants transport more heavy metals to the shoots, reducing root damage.

**Table 1:** Concentration of As, Cd, and Pb in rapeseed plants from field experiment.

Place	Sample	heavy metals	F37	F823	F868	ZY17	FM01
FQ	Root	As	12.83 ± 0.58	10.18 ± 0.40	10.75 ± 0.30	12.71 ± 2.00	9.43 ± 0.25
		Cd	11.23 ± 0.40	7.89 ± 0.29	8.71 ± 0.86	9.48 ± 1.12	6.92 ± 0.21
		Pb	3.82 ± 0.42	2.12 ± 0.21	3.16 ± 1.91	3.77 ± 1.39	1.78 ± 0.26
	Leave	As	14.74 ± 0.16	15.47 ± 0.23	15.83 ± 1.03	14.3 ± 0.20	9.28 ± 0.54
		Cd	9.96 ± 0.19	9.7 ± 0.18	10.03 ± 0.04	9.35 ± 0.01	6.88 ± 0.34
		Pb	3.93 ± 0.27	1.61 ± 0.15	4.78 ± 1.03	0.97 ± 0.31	0.15 ± 1.46
FY	Root	As	9.44 ± 0.41	9.28 ± 0.34	11.13 ± 0.16	8.04 ± 0.35	9.24 ± 0.45
		Cd	7.83 ± 0.04	5.51 ± 0.76	6.26 ± 0.25	5.75 ± 0.12	3.91 ± 0.36
		Pb	1.26 ± 0.54	5.39 ± 1.06	3.02 ± 0.04	3.46 ± 0.33	2.52 ± 0.06
	Leave	As	13.31 ± 0.32	11.66 ± 0.28	14.04 ± 0.55	11.87 ± 0.33	11.37 ± 0.24
		Cd	7.7 ± 0.37	8.2 ± 0.15	10.27 ± 0.30	6.82 ± 0.27	6.28 ± 0.16
		Pb	3.46 ± 0.21	2.01 ± 0.13	3.59 ± 0.43	2.63 ± 0.06	2.62 ± 0.29

### 3.4 Differences in transcription levels caused by heavy metals

**Figure 3:**
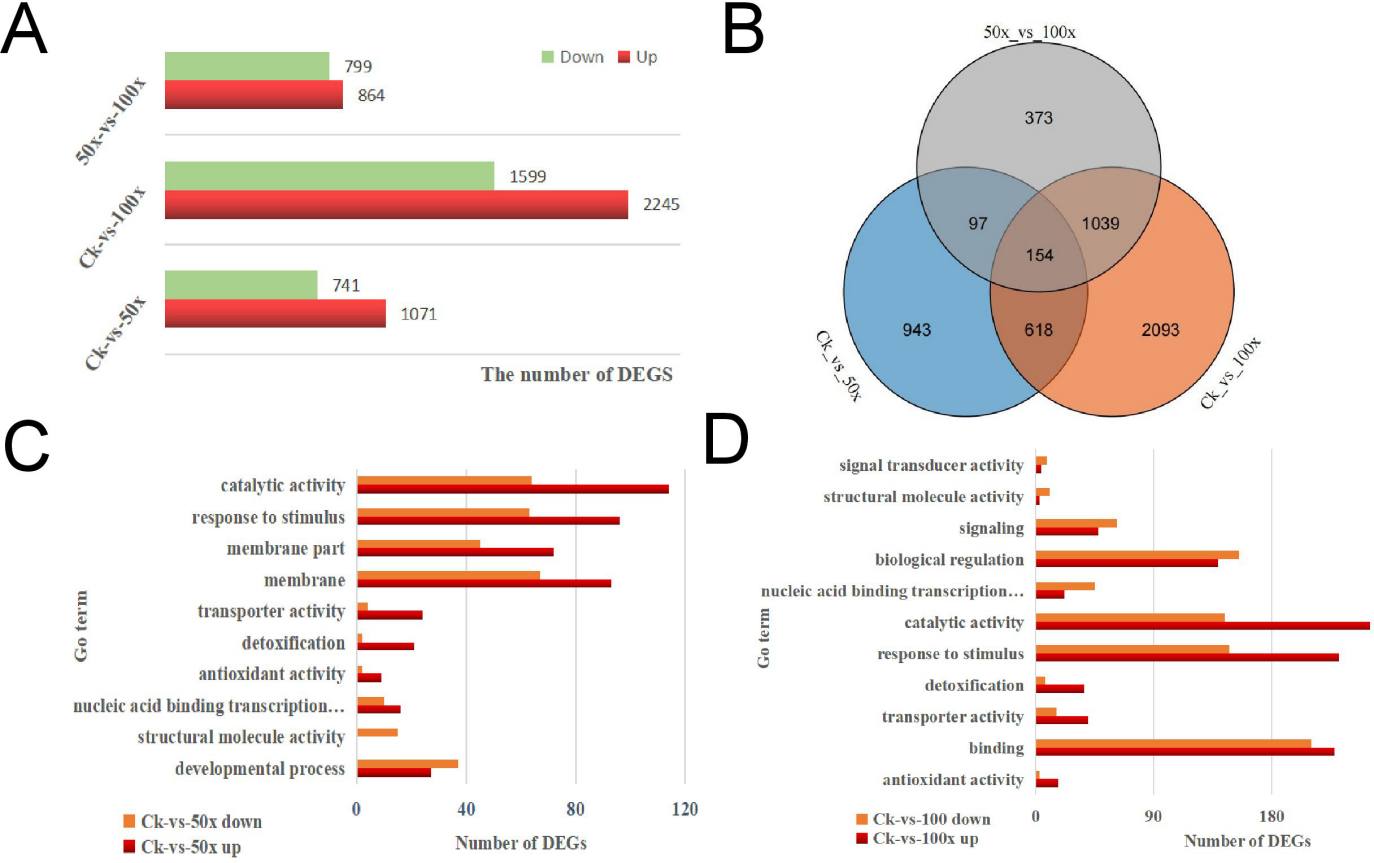
RNA-Seq for WH23 in different treatment groups. **(A)**. the numbers of DEGs in comparison groups; **(B)**. Venn diagram of DEGs; **(C)**. number of DEGs in some important Go/COG pathways; **(D)**. KEGG pathway of DEGs.

There were 1812 (1071 up, 741 down), 3844 (2245 up, 1599 down), and 1663 (864 up, 799 down) DEGs in CK-vs-50×, CK-vs-100×, and 50×-vs-100×, respectively (**Figure 3A**). Only 154 DEGs were co-expressed in three different comparison groups, 943 and 2093 DEGs were identified in CK-vs-50× and CK-vs-100×, respectively (**Figure 3B**). This showed that the gene regulation process of rapeseed differed in response to different levels of heavy metal stress.

GO and COG analyses indicated that rapeseed could effectively enhance catalytic, transporter, and transcription factor activity, regulate the transport and metabolism of inorganic ions, and strengthen antioxidant enzyme activity, immune system, and biological regulation process to improve heavy metals tolerance (**Figure 3C**). However, heavy metals stress also affected the proliferation and differentiation of plant cells, which was not conducive to plant growth and development. When heavy metal stress increased, the nucleic acid binding transcription factor activity, signal transducer activity, and immune system were weakened (**Figure 3D**). This showed that rapeseed has limited ability to regulate heavy metal stress, and that the system will collapse when the stress exceeds a certain level. KEGG analysis indicated that rapeseed could enhance phenylpropanoid, stilbenoid, diarylheptanoid, and gingerol biosynthesis, accelerate amino acid, glutathione, and xenobiotic metabolism, and slow down cell division, to improve heavy metal tolerance; however, there were some differences among different degrees of heavy metal stress (**Supplementary Figure S2**).

### 3.5 Transcription and protein differences in response to heavy metals in different materials

**Figure 4:**
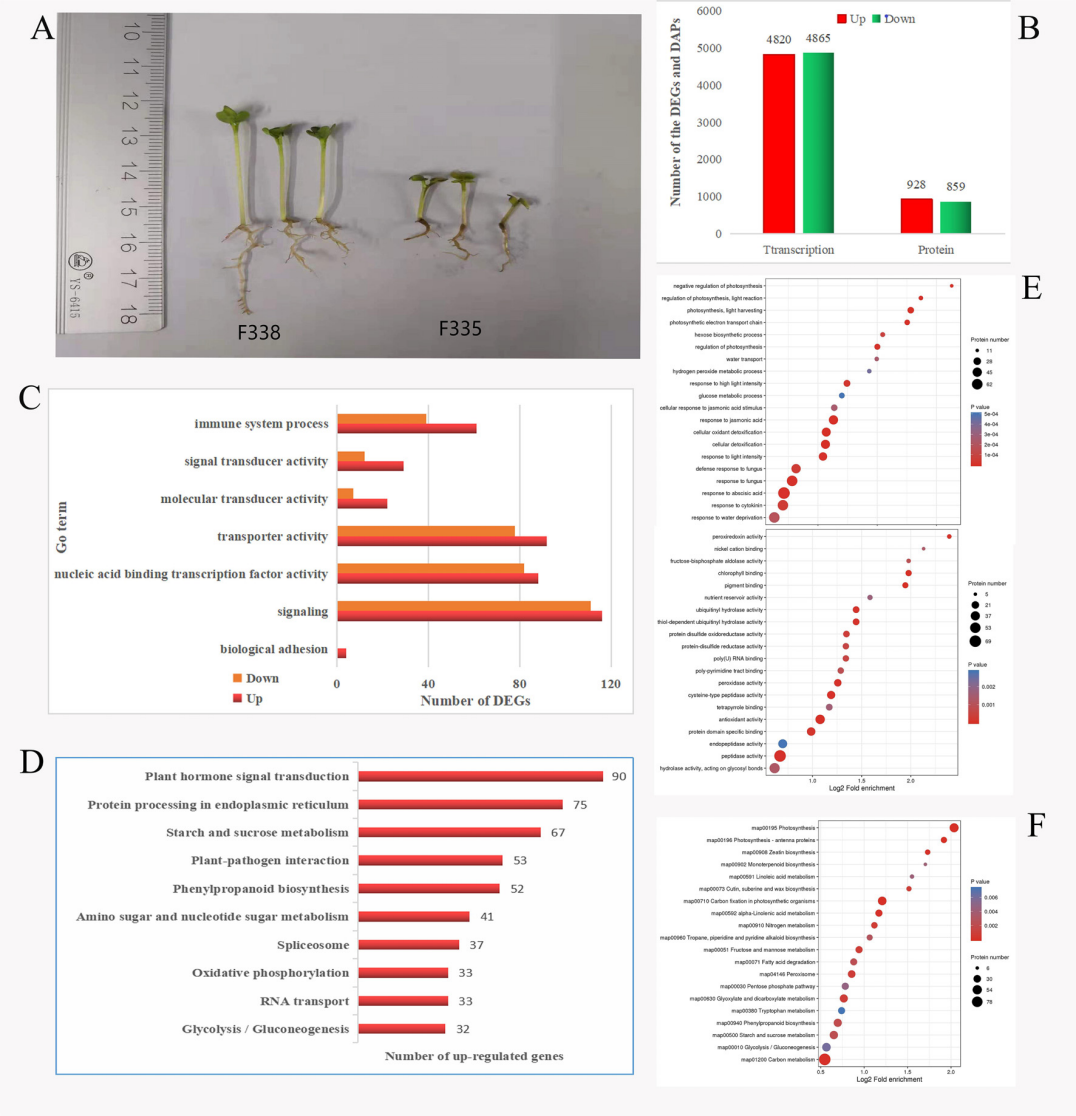
Transcriptome and proteome sequencing of F335 and F338. **(A)** Plants of F335 and F338 in 100x. **(B)** the number of DEGs and DAPs in F335-vs-F338. **(C)** number of DEGs in some important Go/COG pathways. **(D)** the top10 KEGG pathways for up-regulated genes in F335-vs-F338. **(E)** Go analysis for DAPs. **(F)** KEGG analysis for DAPs.

Rapeseed near-isogenic lines (F335 and F338) with significant differences in germination rate in the 100X group were used to analyze the differences in transcription and protein under heavy metal stress (**Figure 4A**). RNA-Seq showed there were 9665 DEGs, with 4820 genes up-regulated and 4865 genes down-regulated (**Figure 4B**). GO and COG analyses showed that F338 had better biological adhesion, transporter, catalytic, and nucleic acid binding transcription factor activity then F335, which may explain why the F338 growth was better in the 100× group than F335 growth (**Figure 4C**). The top 10 KEGG pathways with the most up-regulated genes of F338 compared with F335 were protein processing in endoplasmic reticulum, plant−pathogen interaction, plant hormone signal transduction, RNA transport, starch and sucrose metabolism, spliceosome, cyanoamino acid metabolism, ribosome biogenesis in eukaryotes, ABC transporters, and galactose metabolism (**Figure 4D**). Therefore, from the transcriptome level, F338 could transport heavy metals to vacuoles more rapidly, resulting in improved tolerance. In total, 8926 proteins were identified by quantitative proteome analysis, and 1787 were identified as DAPs, including 928 up-regulated and 859 down-regulated (**Figure 4B**). GO analysis showed that most DAPs in the BP and MF categories were involved in response to hormone and stress, peptidase, and antioxidant activity (**Figure 4E**). KEGG analysis showed that most DAPs were involved in carbon metabolism, carbon fixation in photosynthetic organisms, and photosynthesis pathways (**Figure 4F**). Therefore, from the protein level, the main causes of the phenotypic difference between F338 and F335 in 100× were differences in photosynthesis and plant hormones.

**Figure 5:**
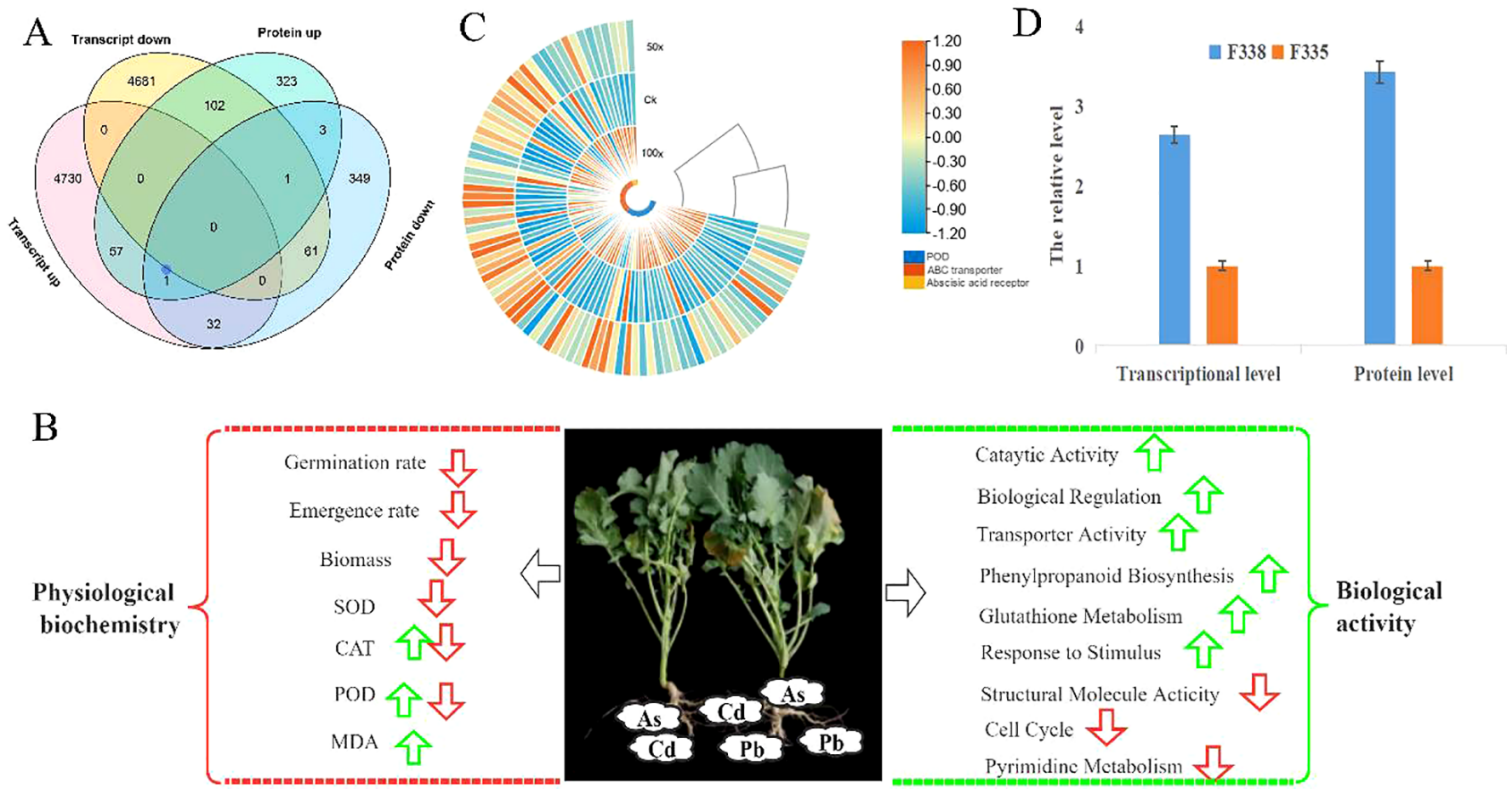
Response mechanism of rapeseed to heavy metal stress based on transcriptome and proteome combination analysis. **(A)**. Vean diagram of DEGs and DAPs in F335-VS-F338. **(B)**. Response mechanism of rapeseed to heavy metal stress. The red arrows were down, green arrows were up, the CAT and POD had different changes in different degree of heavy metals stress, and all the changes were relative to Ck; **(C)**. the expression level of the DEGs related to ABC transports **(D)**. The expression level of some important DEGs.

49162 transcripts and 8925 proteins were quantified by transcriptome and proteome quantification, and 3776 genes were quantified at both transcriptome and proteome levels. There was a weak correlation between transcriptome and proteome, and 118 genes showed the same trend at protein and transcription levels, with 57 genes up-regulated and 61 genes down-regulated (**Figure 5A**); however, 134 genes showed the opposite. The results showed that rapeseed had different regulation of transcription, translation, and post-translation levels to response to heavy metals stress. We focused on the 118 genes with the same trend at protein and transcription levels. GO analysis found that the 118 genes were mainly involved in vacuole, peroxisome, and enzymatic activity, which were also important ways in which plants responded to heavy metal stress. KEGG analysis showed the 118 gene mainly involved in metabiliam process, such as amino sugar and nucleotide sugar metabolism, starch and sucrose metabolism, and beta-Alanine metabolism. Transcriptome and proteome analysis showed that different rapeseed varieties had differences in transcription, translation, and post-translation regulation in response to heavy metal stress, mainly in metabolic process, enzyme activity, and signal transduction.

### 3.6 Molecular mechanism of rapeseed response to heavy metal stress

**Figure 6:**
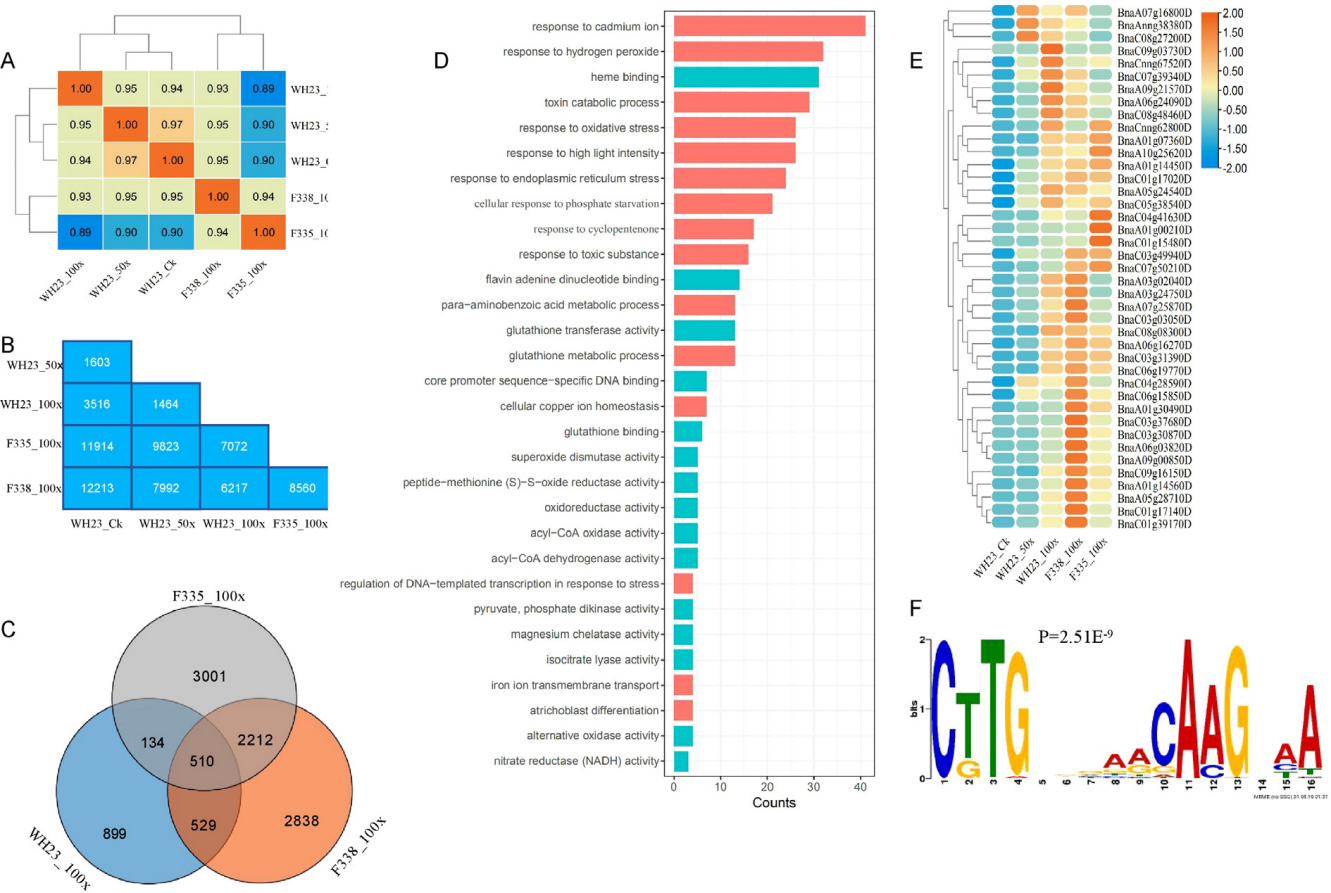
Molecular mechanism of rapeseed response to heavy metal stress. **(A)**. Correlation heatmap of different materials; **(B)**. The number of differentially expressed genes in different comparison groups; **(C)**. Venn diagram of up-regulated gene compared to WH23_Ck; **(D)**. The top 20 GO terms of molecular function and biological process; **(E)** The heatmap of response to cadmium ion; **(F)** The motif of BnaC07g13550D.

WH23 and the rapeseed near isogenic lines (F335 and F338) were used as materials to analyze the common molecular mechanism of heavy metal stress. We obtained a total of 15 high quality transcriptome sequencing data, including WH23 control group treatment (WH23_CK), WH23 50 times concentration treatment (WH23_50x), WH23 100 times concentration treatment (WH23_100x), F335 100 times concentration treatment (F335_100x) and F338 100 times concentration treatment (F338_100x), each treatment had three replicates. The correlation analysis revealed that the correlation between WH23_50x and WH23_CK was the strongest (**Figure 6A**). And the correlation coefficient between F335 and F338 was 0.94, indicating that of the two near isogenic lines varieties may have different response mechanisms under heavy metal stress (**Figure 6A**), this was further confirmed during differential expression genes analysis, there were a total of 8560 DEGs in F338_100x compared with F335_100x, including 4298 up-regulated genes and 4262 down-regulated genes (**Figure 6B**, **Supplementary Figure S2A**). There were most number of DEGs in F338_100x compared with WH23_CK (12213), and the least number of DEGs in WH23_100x compared with WH23_50x (1464) (**Figure 6B**). This showed that the gene regulation process of rapeseed differed in response to different levels of heavy metal stress. Compared with WH23_50x, there were more DEGs in compared with WH23_CK (**Figure 6B**). The above results indicated that both heavy metal stress and the degree of stress could cause changes in the molecular expression levels of rapeseed.

Compared with WH23_CK, there were 2071, 5857, 6089 up-regulated genes in WH23_100x, F335_100x and F338_100x respectively (**Figure 6C**). In order to avoid the differential expression of genes caused by different varieties, we focused on genes that were up-regulated in all three comparison groups. Only 510 genes were found to be up-regulated in all three comparison groups (**Figure 6C**), Go enrichment analysis revealed these 510 up-regulated genes are mainly associated with non-biological stress responses and enzyme activity (**Figure 6D**), including response to cadmium ion, response to hydrogen peroxide, response to oxidative stress and some pathways related to enzyme activity (**Figure 6D**). There were 41 genes related to response to cadmium ion, their expression levels were significantly up-regulated when subjected to heavy metal stress, regardless of different varieties of rapeseed or different degrees of heavy metal stress (**Figure 6E**). Transcription factors played a crucial regulatory role in gene expression levels. Therefore, we analyzed the regulation of these 510 up-regulated genes by which transcription factors. There were a total of 31 transcription factors motifs were significant enrichment in these up-regulated gens promoter sequence (**Supplementary Table S4**) (**Supplementary Figure S2B**), including 6 basic Leucine Zipper Transcription Factor members (bZIP), 9 WRKY DNA-binding domain-containing transcription factor members (WRKY), 8 NAM、ATAF1/2、CUC1/2 transcription factor members (NAC) and some others (**Supplementary Figure S2C**). We noticed that BnaC07g13550D, an NCA transcription factor member, was present in 510 genes, and its motif was significantly enriched in the promoter sequences of these 510 genes (**Figures 6E, F**). It is the homologous gene of *AtNAC13*, which is positive regulation of cellular response to oxidative stress. Perhaps this NAC transcription factor plays an important role in regulating oxidative stress in rapeseed under heavy metal stress. As for down-regulated compared with WH23_CK, there were 584 in all three comparison groups (**Supplementary Figure S2D**). Go enrichment analysis revealed these 584 down-regulated genes are mainly associated withbiological stress responses and cell proliferation, including response to chitin, response to funfgus, cell populationp roliferation, and etc (**Supplementary Figure S2E**). Interestingly, histone H3K9 methylation was significantly enriched in down-regulated genes (**Supplementary Figure S2E**). Histone H3K9 methylation belonged to a type of epigenetic modification, which plays an important role in gene expression regulation, DNA damage repair, and stress resistance.

**Figure 7:**
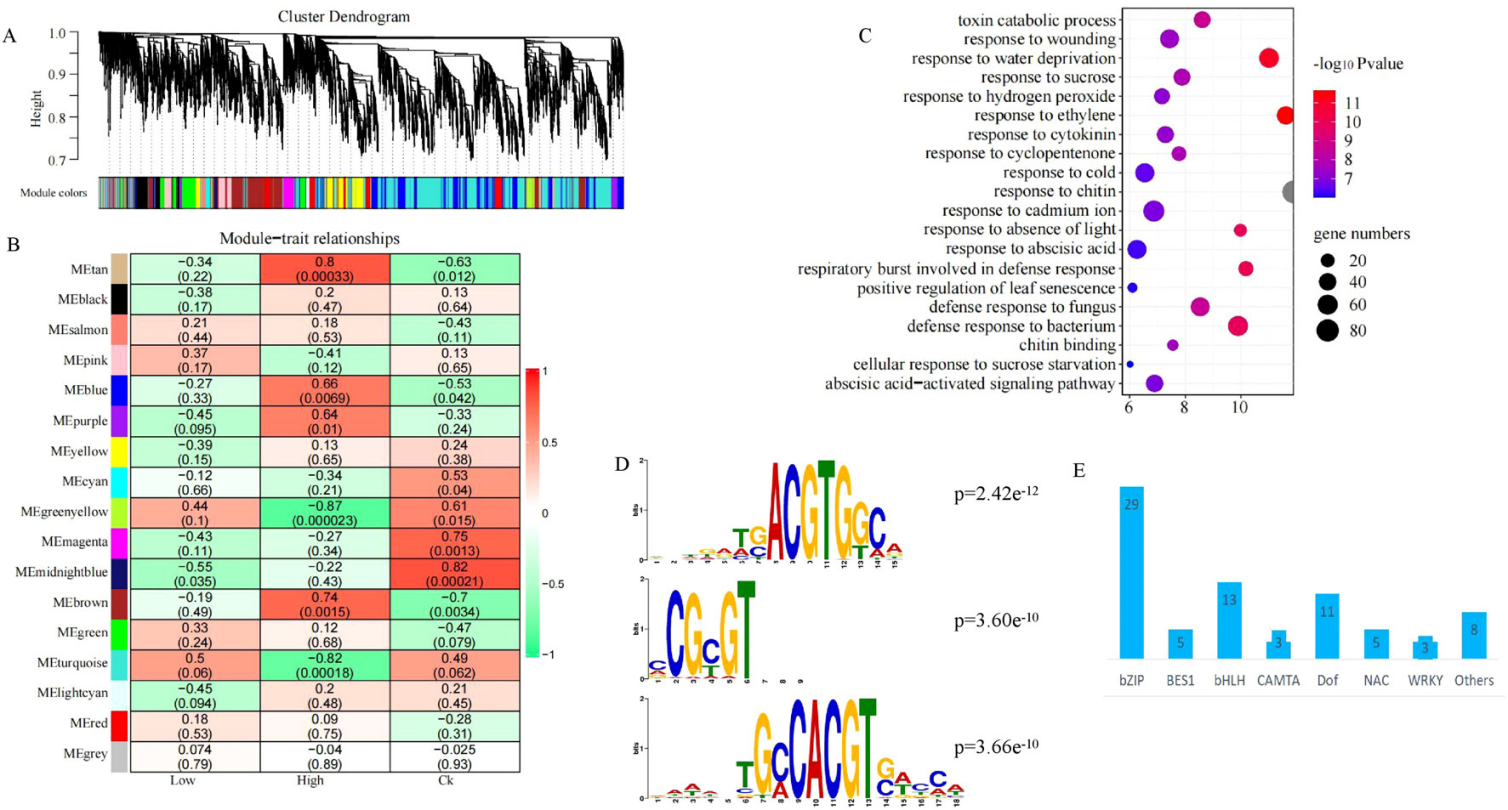
Regulatory network of rapeseed response to heavy metal stress **(A).** Hierarchical cluster tree showing co-expression modules identified using WGCNA; **(B).** Heatmap showed the modules associated with heavy metal stress; **(C)**. The top 20 GO terms of the genes in tan and brown modules; **(D)**. The top 3 motif which were significant enrichment in tan and brown modules. **(E)**. The number of different transcription factor family.

There were too much DEGs in different treatment and varieties, and the overlap DEGs in different compared groups was less. To elucidate the conservative regulatory network of rapeseed in response to heavy metal stress, we selected a set of genes with high variations in different materials for WGCNA. Based on the scale-free network model, 9000 genes were divided into 17 different modules, and the biggest modules have 2709 genes (turquoise module) (**Figure 7A**). Two modules, brown and tan module, were significantly positively associated with high-level heavy metal stress (**Figure 7B**). There were 143 and 1196 genes in tan and brown modules, and these genes were significantly enriched in non-biological stress and hormone response pathways, such as response to cadmium ion, response to abscisic acid, response to ethylene and response to cytokinin (**Figure 7C**). Plant hormones play crucial roles in plant growth, development, and stress response, abscisic acid and sbscisic acid response factor have been confirmed to be associated with plant tolerance to heavy metal stress.

**Figure 8:**
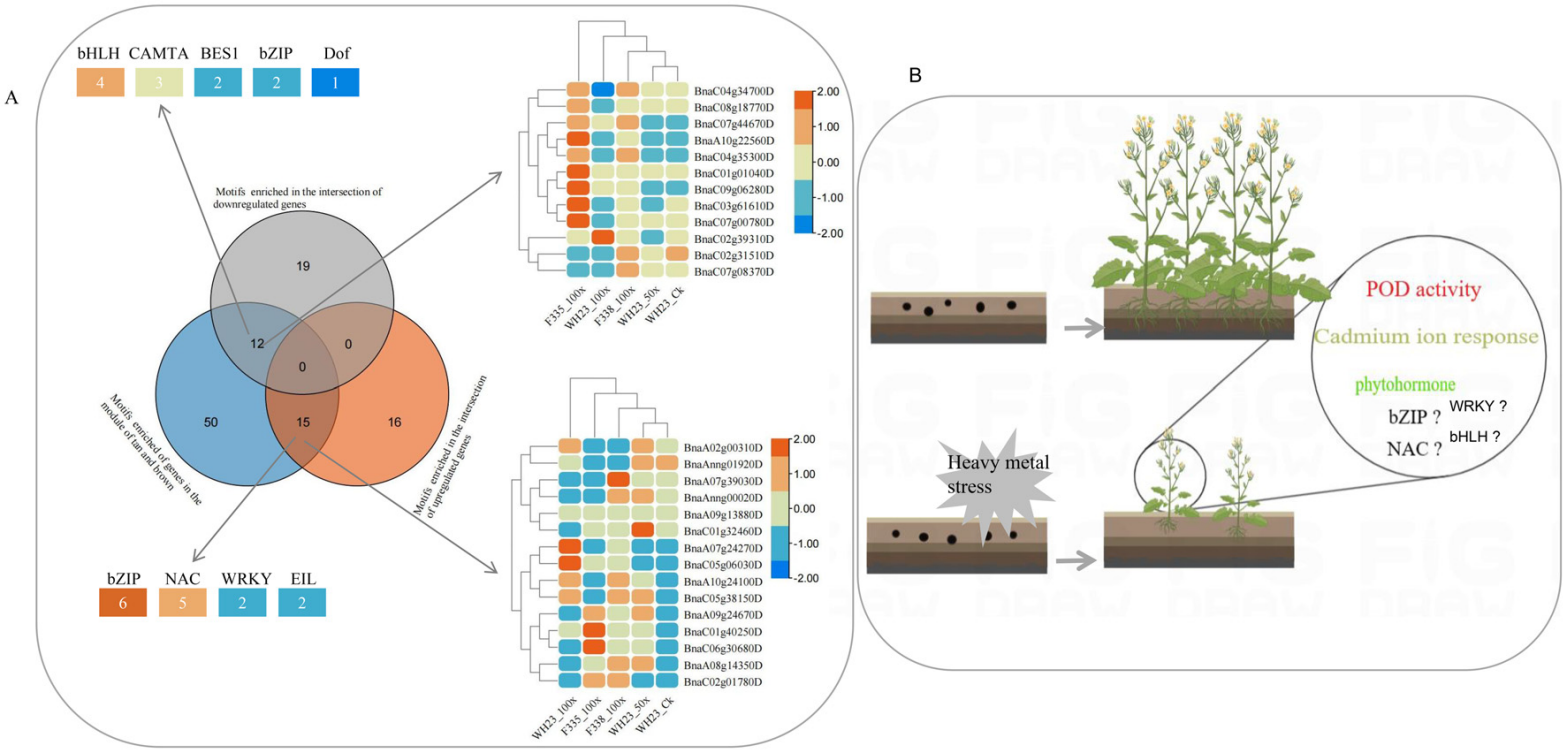
Conservative mechanism of rapeseed respond to heavy metal stress. **(A)**. Venn diagram showed significant enrichment of motifs in different gene sets. The top left and bottom left graphs showed the number of different transcription factor families, and the top right and botton right graphs showed the expression level of different transcription factor; **(B)**. Response mechanism of rapeseed to heavy metal stress.

There were 77 motifs were significant enrichment in the promoter sequence of the genes in tan and brown module (**Supplementary Table S5**), and the top 3 were *BnaA09g24670D*, *BnaC04g34700D* and *BnaC06g00420D*, two of which belonged to bZIP (**Figure 7D**), there were also most member of bZIP (**Figure 7E**). A total of 15 motifs, which were significant enrichment in the promoter sequence of the intersection of up-regulated genes and the WGCNA correlation module genes (**Figure 8A**). These 15 motifs consisted of 6 bZIP, 5 NAC, 2 WRKY and 2 EIL, and most of them were highly expressed when rapeseed is subjected to heavy metal stress (**Figure 8A**). Interestingly, there were no motif significant enrichment in the promoter sequence of the intersection of up-regulated and down-regulated genes (**Figure 8A**). In other words, the intersection of up-regulated and down-regulated genes were not regulated by the same transcription factor.

### 3.7 Mechanism of rapeseed response to heavy metal stress

Combined with the growth phenotype, enzyme activity, sequencing results and bioinformatics analysis of different rapeseed varieties under in different levels of heavy metal stress, we tried to analyze the conservative response mechanism of rapeseed to heavy metal stress. As shown in **Figure 8B**, heavy metal stress first affected rapeseed seed germination and emergence. Although different rapeseed varieties have different performances under the same stress, heavy metal stress is unfavorable to rapeseed germination and emergence, and this conclusion is more obvious under high concentration stress. In indoor experiments and field experiments, we found that the biomass of heavy metal treatment group decreased significantly. When rapeseed was under heavy metal stress, heavy metal ions were absorbed into plants through rapeseed roots, causing toxicity to rapeseed plants, causing oxidative stress and cell membrane damage, and then transported into leaves with rapeseed, thus affecting plant growth and photosynthesis, resulting in decreased biomass. Different from other crops, rape resists heavy metal stress by activating POD and CAT activities, but not SOD (**Figure 5B**). However, this mechanism has its limitations. When the concentration of heavy metals exceeds the critical value, the activities of POD and CAT will also decrease. Rapeseed can activate catalytic and transport enzymes and transport heavy metals to vacuoles faster, thus reducing the toxicity of heavy metal ions. The result also showed that improving the transport capacity of heavy metals in rapeseed plays an important role in enhancing resistance.

At the same time, rapeseed will respond to heavy metal stress by increasing the expression of genes related to biological regulation and stimulation, such as increasing the biosynthesis of phenylpropanoid and the metabolism of glutathione, which are essential substances for plants to respond to abiotic stress. Of course, heavy metal stress will also adversely affect some biological activities of rapeseed. heavy metals will affect the cell cycle and the expression of genes related to pyrimidine metabolism, thus slowing down the growth and development of rapeseed. This may be one of the reasons for the decrease of biomass under heavy metal stress. In addition, heavy metal stress will reduce the activities of photosynthetic enzymes and structural molecules in rapeseed, thus to some extent, plants can not be harmed and develop slowly. In the study, we also found that rapeseed is most sensitive to Cd stress when it faces complex and diverse heavy metal stresses, and As and Pb may have antagonistic effects on the accumulation of heavy metal ions. Cadmium ion response pathway is an important way for rapeseed to respond to heavy metal stress, which will be activated to resist heavy metal stress. As for transcription factors, we think that the bZIP family, NAC family and etc play an important regulatory role.

To sum up, rapeseed can activate antioxidant enzymes and plant gene expression in response to heavy metal stress to resist heavy metal toxicity.

## 4 Discussion

### 4.1 Rapeseed as a potential material for heavy metal phytoremediation

Phytoremediation is considered an effective method for remediation of heavy metal pollution in cultivated land (Shen et al., 2022). At present, hundreds of plant species have been considered as phytoremediation materials, and there are many kinds of hyper-accumulator plants, such as *Pteris vittata L*, *Solanum nigrum L*., and *Celosia argentea* Linn (Lu et al., 2022; Rehman et al., 2017; Yu et al., 2020). However, many hyper-accumulator plants have a low growth rate, low biomass production, and low heavy metal tolerance, which limit their effective use on a large scale (Oladoye et al., 2022). In addition, different from other land types, cultivated land needs to ensure sufficient food production to protect the most basic human needs. In 2014, China completed a national survey on soil pollution, which showed that 19.4% of cultivated land exceeded the farmland standard, and that the main pollutants were heavy metals (GB/T 7714-2015). Therefore, effective utilization and restoration of polluted cultivated land is important for China. In this study, the germination rate of 54 rapeseed varieties was > 80% in the 100× group, and the highest heavy metal content was 417.64 µg/g. In the field experiment, the highest heavy metal content without affecting normal rapeseed growth was 53.07 µg/g. In our study, we selected only three heavy metals (Cd, Pb, and As), and rapeseed could simultaneously immobilize these three heavy metals. Previous studies have shown that rapeseed can also immobilize Cu, Zn, and other heavy metals (Munir et al., 2020). In addition, China imports > 70% edible oil every year (China National Bureau of Statistics, http://www.stats.gov.cn/ ), and improving edible oil self-sufficiency rate is one of the national policies. Rapeseed is one of the main oil crops in China, and is grown in winter in Yangtze River basin which is different from the growing season of grain crops. The Yangtze River basin is the main production area of rapeseed; however, it is also polluted by heavy metal (Zhou and Wang, 2019). Therefore, rapeseed use as material for heavy metal phytoremediation can simultaneously realize heavy metal remediation in farmland and increase edible oil production.

### 4.2 DEGs and DAPs involved in antioxidant-system

Many studies have shown that when plants are stressed by heavy metals, the associated excessive ROS production causes serious damage (Yang et al., 2020; Munir et al., 2020). To reduce the cellular damage caused by excess ROS, plants usually activate antioxidant systems to eliminate excess ROS (Huang et al., 2019). SOD, CAT, and POD are important enzymes in plant antioxidant system and are involved in the detoxification of O^2-^ and H_2_O_2_ and inhibition of the formation of OH^-^ radicals. In our study, we found that rapeseed could activate antioxidant enzymes to respond to heavy metal stress, but that when the heavy metal stress was too high, this mechanism would not function, which was similar to the results of Zhang F, et al. (2020). In F338-vs-F335, a total of 23 DAPs were involved in the antioxidant system, including 4 SOD, 3 CAT, and 16 POD (**Supplementary Table S6**). In addition, in RNA-seq, a total of 63 DEGs were involved in the antioxidant system, including 15 SOD, 6 CAT, and 42 POD (**Supplementary Table S6**). The number of DAPs and DEGs involved in POD were more than that involved in SOD and CAT, and the POD activity was also higher than SOD and CAT activity, suggesting that POD plays a more important role in rapeseed response to heavy metal stress. Comparing the expression level of DEGs involved in POD, the expression levels in the 50× and 100× groups were higher than that in CK, which also showed that rapeseed could activate POD activity in response to heavy metal stress. Therefore, if POD activity can be controlled by a modern molecular biology technique, the tolerance of rapeseed to heavy metal stress can be improved, and rapeseed can even be used for phytoremediation of heavy metal contaminated cultivated land.

### 4.3 DEGs and DAPs involved in heavy metal transport

Plant roots can absorb and transport nonessential and toxic heavy metal ions via transporters for essential metal ions, such as Mn^2+^, Fe^2+^, Cu^2+^, and Zn^2+^ (Chen et al., 2020). ABC transporters, heavy metal ATPases (HMA) family, and zinc−iron permease (ZIP) family are three important transporters in plant, and are important for heavy metal transportation and distribution (Tsednee et al., 2014). In F338-vs-F335, a total of 8 DAPs were involved in heavy metal transporters, including 6 ABC transporters and 2 HMA (**Supplementary Table S4**), and all HMA were down-regulated. In RNA-Seq, we identified a total of 57 DEGs involved in heavy metals transporters, including 8 HMA family members, 6 ZIP family members, and 43 ABC transporter (**Supplementary Table S4**). **Figure 5C** shows the expression levels of 43 ABC transporters in the CK, 50×, and 100× group, and that the expression of most of ABC transporters was higher under heavy metal stress. For example, the expression of BnaC04g51510D (ABC transporter) in the 50× and 100× was nearly twice and three times higher, respectively, than that in CK. Interestingly, BnaC04g51510D was also identified as DEG and DAP in F338-vs-F335. At the transcriptional and protein levels, BnaC04g51510D was approximately three times higher in F338 than in F335, which may explain the higher tolerance of F338 to heavy metal stress (**Figure 5D**). In fact, BnaC04g51510D belongs to ABC transporter B family (ABCB) member, and many studies have showed that ABCB could combine and hydrolyze ATP to provide energy and realize the trans-membrane transport of heavy metal ions, inorganic molecules, organic molecules, and other substances (Sánchez-Fernández et al., 2001; Verrier et al., 2008). In plants, the ABC transporter family is one of largest gene families, and is related to plant stress resistance; the number of ABC transporter family members affects plant tolerance to stress. There are 314 ABC transporter family members in rapeseed, more than that in maize, rice, and arabidopsis (Zhang et al., 2018; Guo et al., 2022); this may result in rapeseed being more resistant to stress than these other plant species.

### 4.4 DEGs and DAPs involved in signal transduction

Signal transduction was important for plants to responds to various stimulus; plants have different signal transduction characteristics for different stimuli. Plant hormones and calcium signals are core transducers in many adaptations and developmental processes in plants could be elicited by many abiotic factors such as salt, light, and heavy metal stress (Kudla et al., 2010; Thao et al., 2015; Chen et al., 2020). There were 6 DAPs involved in plant hormones and calcium signals in F338-vs-F335, including 1 gibberellin regulated protein, 1 abscisic acid receptor, and 4 calcium signals associated proteins (**Supplementary Table S4**). Furthermore, in the RNA-Seq, there were 64 DEGs involved in plant hormones and calcium signals, including 14 auxin-responsive, 7 abscisic acid receptor, 12 gibberellin regulated protein, and 31 calcium signaling related genes (**Supplementary Table S4**). Compared to the expression level for 7 abscisic acid receptor DEGs with different heavy metal treated, it is found that heavy metal stress would increase the expression of abscisic acid receptors, and the level of expression increased with the degree of stress (**Figure 5C**). Therefore, rapeseed could respond on heavy metal stress by abscisic acid. Abscisic acid is an important hormone for plants to respond to abiotic and biotic environmental changes (Chen et al., 2020; Hu et al., 2020). heavy metal stress could increase abscisic acid biosynthesis, and abscisic acid could alleviate the harm caused by heavy metal stress (Hu et al., 2020). Therefore, the tolerance of rapeseed to heavy metal stress could be improved by applying exogenous abscisic acid.

### 4.5 Molecular mechanism of rape affecting heavy metal stress

Previous studies have shown that NAC transcription factor family is unique to plants and involves physiological processes of development and stress response (Puranik et al., 2012), which plays an important role in plant resistance to abiotic and biological stresses (Cui et al., 2021). found that NAC46 can positively regulate allergic response, which is a transcription activator and a positive regulator of cell death and chlorophyll degradation. In our research, it was found that BnaC07g13550D, a member of NCA transcription factor, existed in 510 up-regulated genes of rape against heavy metal stress, and its motifs were significantly enriched in the promoter sequences of these 510 genes, which were homologous to AtNAC13, the positive regulation of cell response to oxidative stress, indicating that in the process of rape against heavy metal stress, it is possible to enhance the tolerance of plant cells to heavy metal stress and enhance rapeseed resistance by enhancing the expression of BnaC07g13550D.Energy homeostasis is very important for all stages of plant life. Plants suffer from abiotic stress, which leads to senescence or ROS accumulation. By activating or regulating related genes, they can resist and maintain life homeostasis. Studies have shown that bZIP is associated with stress response, cell cycle regulation and various developmental aspects.bZIP1 was found to be involved in the inhibition of metabolic signal glucose (Kang et al., 2010), bZIP11 could control auxin to promote the growth of primary roots (Weiste et al., 2017), bZIP10 and bZIP25 formed dimers with bZIP53 to activate seed maturation genes (Alonso et al., 2009), bZIP29 was found to change the number of leaf cells and the development of root meristem function (Leene et al., 2016), and bZIP59 was defined to play an important role in auxin-induced callus formation and plant regeneration (Xu et al., 2018). Howell (2013) found that bZIP can reduce misfolded proteins in plants under adverse environment, and over-expression of bZIP17 can enhance the tolerance of plants to salt treatment (Liu et al., 2008). BZIP19 and bZIP23 have been proved to control the genes encoding Zn transporters and promote the adaptation of plants to zinc (Assuncao et al., 2010). In our experiment, it was found that when rapeseed was subjected to heavy metal stress, the bZIP motif in the promoter sequence of the module gene was significantly enriched, and most of them were highly expressed. At the same time, the responses to abscisic acid and cytokinin were also significantly enriched in abiotic stress and hormone response pathways. Furthermore, it shows that transcription factors such as bZIP may be induced by heavy metal stress, activated after ABA signal transcription, and implemented adaptive response to counteract heavy metal toxicity in cell tissues (Banerjee and Roychoudhury, 2015). In the study, it was also found that rape was most sensitive to Cd stress when faced with complex and diverse heavy metal stresses, and as and Pb might have antagonistic effects on the accumulation of heavy metal ions.

## 5 Conclusion

Rapeseed has great potential in phytoremediation of heavy metal polluted arable land; however, the molecular basis of heavy metal tolerance in rapeseed is largely unclear. In this study, we treated 127 different varieties of rapeseed with different concentrations of heavy metals (Cd, Pb, and As), transcriptome sequencing for different treated materials (CK, 50×, and 100×), and transcriptome and proteome sequencing of different materials (F338 and F335). Combined with these results, heavy metals can adversely affect the emergence and seedling growth of rapeseed. Rapeseed mitigates the harm of heavy metal stress by activating the enzymatic activity of the antioxidant enzyme system and the heavy metal transport system, and peroxidase, ABC transporter, and abscisic acid play an important role in these mechanisms. In this study, we propose a potential phytoremediation material for heavy metal contamination in cultivated land (WH23) and provide new insights into the molecular mechanism of heavy metal tolerance in rapeseed.

## Data Availability

The datasets presented in this study can be found in online repositories. The names of the repository/repositories and accession number(s) can be found in the article/**Supplementary Material.**

## Supplementary material

Supplementary Figure 1 The activity of CAT and SOD in two different experimental field.

Supplementary Figure 2 Changes in molecular functions of rapeseed in response to heavy metal stress. **(A)** The number of down-regulated genes in different comparison groups; **(B)** The top 3 motif which were significant enrichment in up-regulated genes; **(C)** The number of different transcription factor family; **(D)** Venn diagram of down-regulated gene compared to WH23_CK; **(E)** The top 20 GO terms of molecular function and biological process.
