# Supplementary figures and images for: Conservative mechanism through various rapeseed (Brassica napus L.) varieties respond to heavy metal (Cadmium, Lead, Arsenic) stress
Source: Front Plant Sci. 2025 Jan 14;15:1521075. doi: 10.3389/fpls.2024.1521075 (PMC11773377; doi:10.3389/fpls.2024.1521075)

The activate of SOD and CAT

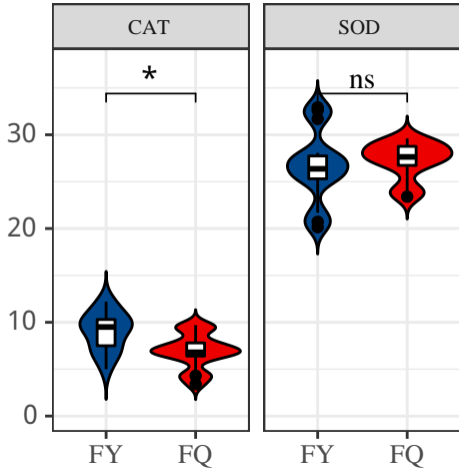

Supplement: Supplementary Figure 1 — The activity of CAT and SOD in two different experimental field. [file Image1.pdf]

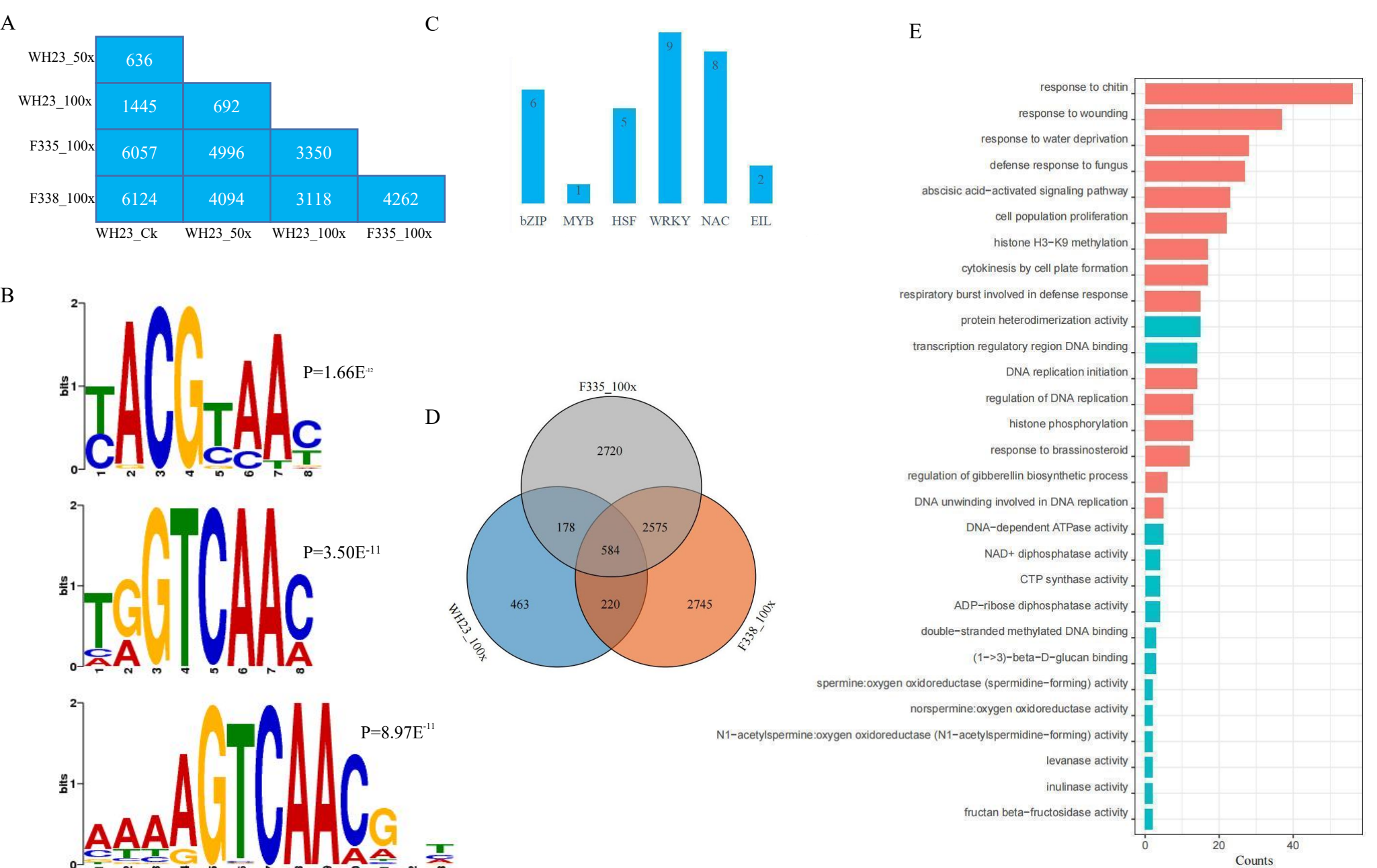

Supplement: Supplementary Figure 2 — Changes in molecular functions of rapeseed in response to heavy metal stress. (A) The number of down-regulated genes in different comparison groups; (B) The top 3 motif which were significant enrichment in up-regulated genes; (C) The number of different transcription factor family; (D) Venn diagram of down-regulated gene compared to WH23_CK; (E) The top 20 GO terms of molecular function and biological process. [file Image2.pdf]
